# See Salt: Recommendations for engaging oyster growers in community-based coastal monitoring programs

**DOI:** 10.1007/s10661-025-14560-y

**Published:** 2025-09-11

**Authors:** Natalie G. Nelson, Marcelo Ardón, Tal Ben-Horin, Eric Herbst, Whitney Knollenberg, María Menchú-Maldonado, Christopher L. Osburn

**Affiliations:** 1 Biological and Agricultural Engineering, North Carolina State University, Raleigh, NC USA; 2 Center for Geospatial Analytics, North Carolina State University, Campus Box 7625, Raleigh, NC 27695 USA; 3 Forestry and Environmental Resources, North Carolina State University, Raleigh, NC USA; 4 College of Veterinary Medicine, North Carolina State University, Morehead City, NC USA; 5 North Carolina Sea Grant, Morehead City, NC USA; 6 Parks, Recreation, and Tourism Management, North Carolina State University, Raleigh, NC USA; 7 Marine, Earth, and Atmospheric Sciences, North Carolina State University, Raleigh, NC USA

**Keywords:** Citizen science, Mariculture, Salinity, Water level, Participatory science

## Abstract

Aquatic monitoring of estuarine and coastal waters remains limited due to steep maintenance requirements for in situ hardware stemming from rapid biofouling and harsh environmental conditions. Monitoring sites will also ideally span a mix of shoreline and open water locations, and the need to travel to remote locations can strain resources and limit the geographic footprint of coastal monitoring programs. To overcome these limitations, we explored opportunities to partner with aquatic industry professionals who work on the water and may be able to incorporate routine site visits for sensor maintenance as part of their day-to-day routines. We specifically focused on collaborating with oyster growers to collect continuous salinity and water level observations on their farms, as part of a community-enabled monitoring program we named “See Salt.” In this short communication, we present the See Salt program as a case study and provide recommendations for others who may be interested in establishing a similar community-supported coastal monitoring program. Our recommendations include the following: (1) establish multi-method evaluation strategies to gain insights on program improvement, (2) invest in hardware that can go long durations without recalibration and cleaning, and (3) provide participants with the equipment and training needed to maintain sensors and validate measurements on their own. While we focus on our experiences monitoring salinity and water levels with oyster growers, the recommendations are not specific or unique to these variables or this partner population; our findings are broadly applicable to aquatic monitoring performed in partnership with non-academic professionals.

**Supplementary information:**

The online version contains supplementary material available at 10.1007/s10661-025-14560-y.

## Introduction

Salinity is one of the most important determinants of life and loss at the coast. In estuaries and coastal areas, fluctuating salinities driven by rainfall, runoff, and tides create ranging habitat types that support many different species, creating biodiversity hotspots (Kennish, 2002). However, salinity can also lead to consequential impacts to infrastructure and natural resources (Hummel et al., 2018; Tansel & Zhang, 2022; Tully et al., 2019). When salt water infiltrates into usually fresh waters, communities experience loss of crops and trees, contamination of drinking water supplies, wastewater system failures, and metal corrosion in infrastructure.

Despite the importance of salinity for both natural and built environments, very little continuous monitoring infrastructure exists to detect, report, and predict changes in salinity in estuarine and coastal waters. The United States (U.S.) Geological Survey launched the Coastal Salinity Index (CSI) project in 2019 (Conrads & Darby, 2017), through which they compiled the best available real-time salinity data sources on the Atlantic and Gulf coasts. The CSI is among the most comprehensive salinity monitoring initiatives in the USA to date, yet it only includes 130 stations for tens of thousands of miles of tidal coastline. The U.S. National Estuarine Research Reserve System also maintains a System Wide Monitoring Program, through which they monitor many variables, including salinity, with high-frequency multiparameter sondes across multiple sites within each of their reserves (Mills et al., 2008; Porter et al., 2004). While some sites transmit data in near real-time, data from the remaining non-telemetered sites are uploaded to a publicly accessible repository approximately monthly. The lack of high-frequency and real-time monitoring is particularly concerning in the context of accelerating sea level rise (Piecuch & Hamlington, 2023), which will fundamentally change coastal salinity dynamics (Helton et al., 2025). Basic questions on rates of salinity change and its effects on ecosystems and infrastructure are all constrained by lack of data coverage.

Salinity is a straightforward variable to compute using continuous and in situ conductivity and temperature sensors, which are widely available from commercial environmental monitoring companies, but their steep maintenance requirements in coastal and estuarine waters create barriers to their expanded deployment. Because coastal waters are home to quick-growing plants and animals (e.g., algae, barnacles), instruments are prone to “biofouling” over short time periods (e.g., under a week; Delauney et al., 2010) and must be regularly cleaned and calibrated (Koren & McGraw, 2023). In a survey of 145 coastal practitioners and researchers, biofouling and associated maintenance were reported as the primary issues users faced when working with conductivity and temperature sensors (Alliance for Coastal Technologies, 2007). Moreover, salinity monitoring stations will ideally be located across a mix of shoreline and open water locations, which requires time and resources to boat to remote locations. Government offices tasked with aquatic monitoring often lack the funding or personnel needed to support such efforts. High precision instruments with the capacity for long-term deployment can also be expensive (> $5,000 USD), which further limits widespread deployment of sensor networks.

Community-enabled monitoring could serve as an approach for overcoming maintenance barriers preventing widespread salinity monitoring, as evidenced by the success of other community-supported environmental monitoring programs like the Community Collaborative Rain, Hail, and Snow (CoCoRaHS) network (Reges et al., 2016; CoCoRaHS (n.d.), a volunteer-based weather monitoring program. Because precipitation falls everywhere, the CoCoRaHS network can engage wide-ranging audiences. In contrast, fewer options exist for community partnerships to support coastal salinity monitoring, but there are diverse professionals who work on the water and may be able to incorporate routine site visits for sensor maintenance as part of their day-to-day routines. For example, mariculturists (e.g., shellfish growers) regularly visit their farms to tend to their product. If salinity monitoring equipment was installed on facilities such as oyster farms, growers could check on equipment and clean it of biofouling during routine visits, thus overcoming the barrier of needing dedicated personnel and transportation for maintenance. Moreover, oyster growers may have incentive to collect salinity data on their farms, as salinity is directly related to oyster quality (e.g., La Peyre et al., 2013), and a predictor of economic loss (e.g., Evans et al., 2016). An oyster grower-enabled salinity monitoring network could create opportunities to alleviate maintenance burdens associated with spatially extensive salinity monitoring, while also creating a path forward for data-driven precision mariculture. While land-based agriculture has benefitted from access to a growing number of precision and data-driven technologies, relatively fewer precision tools exist to support mariculture operations. To our knowledge, partnering with oyster farmers to expand salinity monitoring has not previously been piloted. Oyster farmers have, however, participated in community-supported monitoring programs, such as by filming submerged cages with cameras to document habitat provisioning for fish (Mercaldo-Allen et al., 2021).

## See Salt: A salinity and water level monitoring program in partnership with oyster farmers

In 2024, we created and piloted “See Salt,” an oyster-grower enabled salinity monitoring program to obtain continuous salinity and water level observations on estuarine oyster farms in North Carolina, USA. Our goal was to establish a framework for generating real-time salinity data that fills spatial and temporal monitoring gaps, supports multiple applications and research areas, and is mutually beneficial to data collectors and users. In this short communication, we present the See Salt program as a case study and provide recommendations for others who may be interested in establishing a similar community-supported coastal water quality monitoring program based on our lessons learned. Prior calls to action have requested such case studies be presented in support of developing effective practices for community-based environmental monitoring (Conrad & Hilchey, 2011). While we focus on our experiences monitoring salinity and water levels with oyster growers, the recommendations are not specific or unique to these variables or partner population; our findings are broadly applicable to aquatic monitoring performed in partnership with non-academic professionals.

**Fig. 1:**
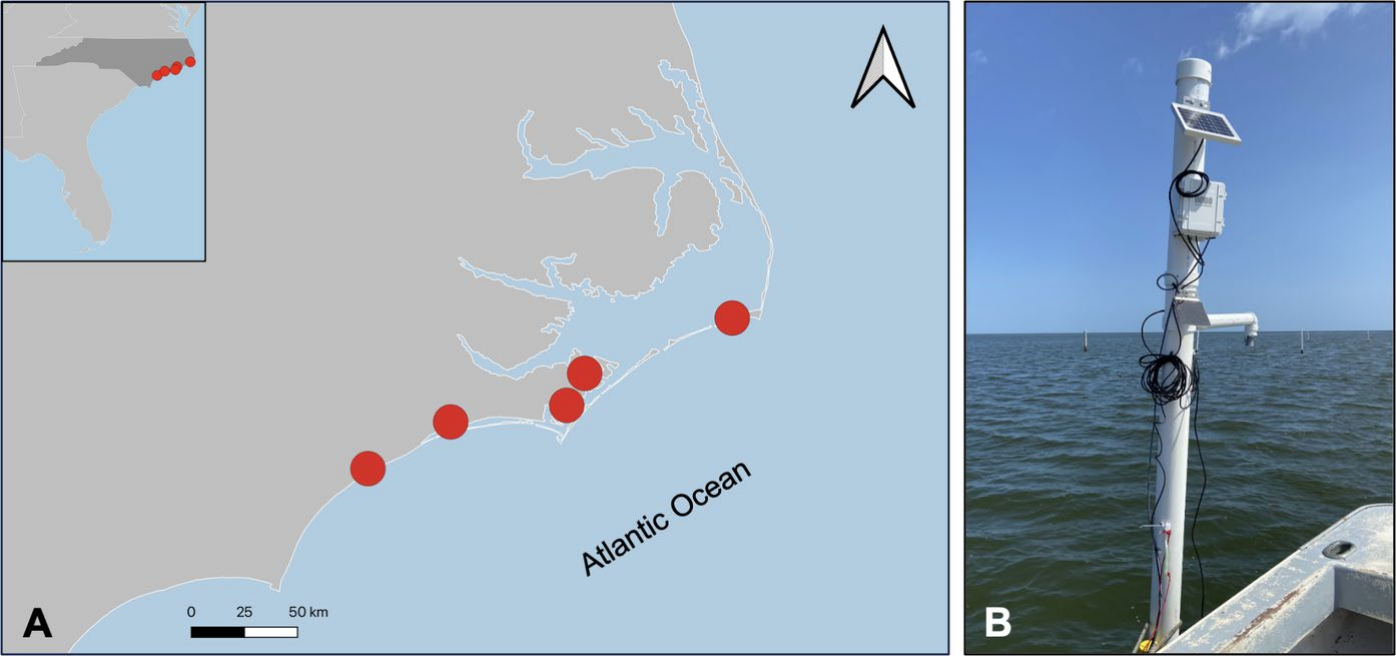
**A** See Salt pilot program participant locations across five estuarine waterways in North Carolina, USA. **B** Photograph of monitoring units installed at one site; the ultrasonic sensor is included at the end of the arm coming out of the PVC post. Photo taken by Natalie Nelson

As part of the pilot, we partnered with five commercial oyster farming operations in North Carolina, which were located across diverse estuarine waterbodies (Fig. 1); these operations were not affiliated with our institutions. Salinity and water level observations were collected continuously (i.e., 1-min and 5-min temporal resolution, respectively) for approximately four to 8 months, depending on the site. At each farm, we installed two monitoring units: an industry-standard unit and a low-cost unit. For the industry-standard unit, we monitored conductivity and temperature with a pHionics sensor (STs series, 0–100,000 µS/cm range, 0–50 °C range, 4–20 mA output) and water level with an Onset HOBO sensor (pressure transducer); the sensors were connected to a solar-powered Onset HOBO RX3000 4G remote monitoring station, which allowed for near real-time data transmission. We describe this unit as “industry-standard” because Onset HOBO equipment is commonly used by researchers, including in continuous monitoring networks (e.g., SeagrassNet uses Onset HOBO equipment for continuous light and temperature monitoring; Short et al., 2015). The use of a pHionics sensor with Onset HOBO hardware exists in peer-reviewed literature (Routhier et al., 2024). The low-cost unit, the “Tide Information Monitor” (TIM), was produced by Tidal Eye, LLC, and included a conductivity and temperature sensor, as well as an ultrasonic-based water level sensor; the TIM was also solar-powered and transmitted data in near real-time. The HOBO unit with pHionics sensor and TIM unit cost approximately $3,300 and $1,100, respectively, including data transmission fees.

## Lessons learned and recommendations

Here, we outline lessons learned from our experience piloting See Salt. We delve into the importance of multi-method evaluation strategies to continuously improve programs, the necessity of investing in durable hardware that minimizes recalibration and cleaning frequency, and benefits of equipping and training participants to independently maintain sensors and validate measurements.

### Establish multi-method evaluation strategies to gain insights on program improvement

See Salt was supported by a multi-disciplinary academic team who contributed expertise in the design, deployment, and data analysis of the community-supported salinity monitoring program. A social scientist team member (W. Knollenberg) developed and implemented an interview protocol to evaluate the program through qualitative data gathered from community members (i.e., oyster growers) (NC State Institutional Review Board approval was obtained; protocol 27282; Online Resource 1). The five pilot program members were interviewed to gain feedback on the deployment, maintenance, and usability of the salinity monitoring units. Additional oyster growers were interviewed to gain insights on perceived benefits and challenges of participating in a community-based salinity monitoring program. As seen with the synthesis of qualitative interview data and quantitative monitoring data below, strategies to improve the program were derived by triangulating the interview data, quantitative data gathered through the two salinity monitoring units, and team members’ observations of the monitoring units’ deployment and maintenance procedures. Because these multiple data sources provided a more robust understanding of the challenges (e.g., growers’ perceptions of cleaning and calibration) related to See Salt, for community-supported monitoring projects, we recommend integrating multi-method evaluation strategies such as these to generate robust data that offer multiple perspectives on how to improve or maintain a program’s success.

### Invest in hardware that can go long durations without recalibration and cleaning

**Fig. 2:**
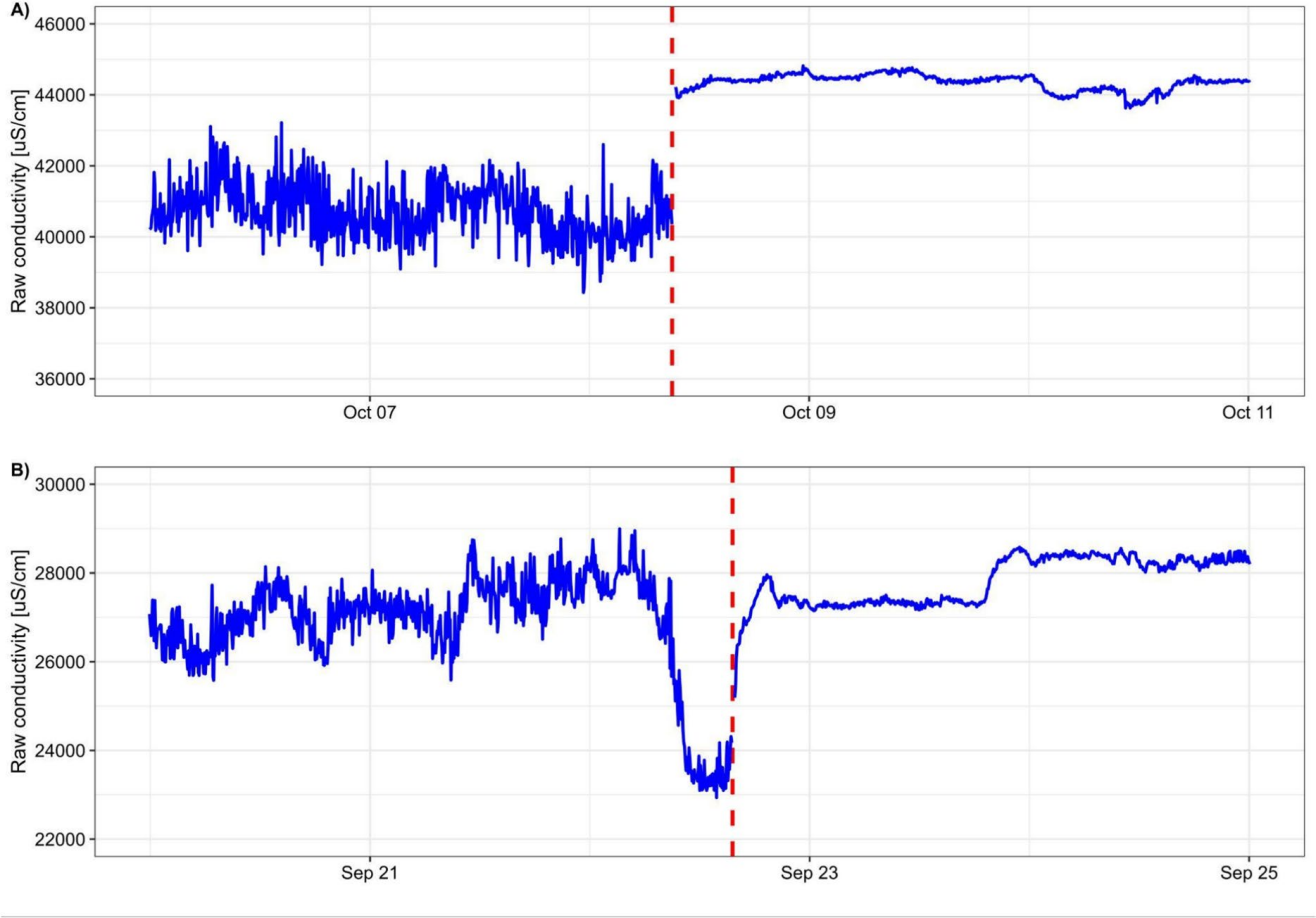
Subsets of raw conductivity time series collected at two different sites (**A**, **B**) with the pHionics sensor installed on the HOBO unit. The red dashed lines correspond to instances when conductivity sensors were cleaned. The conductivity values change in magnitude and variance following cleaning

Though some of our program participants regularly cleaned and maintained the sensors installed on their farms, biofouling remained a challenging issue to overcome, and fouling affected measurements (e.g., see Fig. 2). Cleaning increased the sensitivity of the conductivity sensors as evidenced by increased readings immediately after cleaning (Fig. 2), illustrating the importance of routine maintenance. We also noticed that the variance in conductivity readings decreased after cleaning, and at this point do not know the reason for this pattern. Biofilms can alter sensors in various ways, such as by serving as diffusion barriers, transforming analytes, or blocking access to receptors (Koren & McGraw, 2023). We will continue to work with these sensors to better understand how biofilms affect conductivity measurements.

For a trained user, sensor cleaning and maintenance required approximately 15 min of one person’s time. And some pilot program members recognized the value of this time investment, “It's a commitment. If you want good data, you have to commit to your part” (Pilot Program Member 4). Though 15 min may seem brief, oyster farmers have many time-consuming tasks to complete while on the water, making additional tasks potentially burdensome. Of the farms we partnered with (Pilot Program Member 3), the one that cleaned sensors most reliably had assigned maintenance tasks to an intern; this operation was also land-based and did not need to travel by boat to their farm, which made maintenance tasks less logistically challenging. In their interview, this pilot participant noted that cleaning was not a challenge, “It takes five minutes… Maybe ten. I have one of my employees do it.”

Some monitoring equipment available on the market includes anti-fouling measures, such as wipers that will regularly clean sensor faces. Though equipment with anti-fouling measures is often costly, anti-fouling measures could lengthen the amount of time needed between maintenance visits, and the cost-savings associated with fewer maintenance trips may justify the greater initial capital investment. However, greater initial capital costs also likely affect the number of monitoring units that a program or grant can acquire. From our experience, we expect that community-supported monitoring projects using fewer sites (e.g., “sentinel” sites) to strategically fill gaps in existing monitoring networks are likely more feasible and justifiable than having a more spatially dense network of monitors that are less robust to field conditions.

Issues with rapid fouling, however, were unique to the sensors that remain submerged (i.e., conductivity and temperature sensors, pressure transducer). The ultrasonic water level sensor on the TIM unit remained above the water surface and did not require frequent cleaning. Therefore, from our experience, we see greater opportunities for widespread aquatic monitoring with low-cost sensors in situations where biofouling is readily avoidable. One example might include deploying the TIM unit’s ultrasonic sensor across residential docks to expand water level monitoring.

### Provide participants with the equipment and training needed to maintain sensors and validate measurements on their own

In the See Salt pilot, we sought to minimize the amount of time our participants dedicated to sensor calibration, which we originally estimated would need to occur approximately every other week. Our approach to calibration involved having a team member periodically place the sensors in calibration solutions, the times when the sensors were submerged in solutions were noted, and recalibration was then applied post-hoc. We had also planned to provide participants with calibration solutions, such that they could similarly place the sensors in calibration solutions and notify us of the times in which the sensors were in the solutions. We could then apply an updated calibration on the backend. However, this approach left the participants in the dark in terms of understanding whether the sensors were collecting accurate measurements. In some cases, participants questioned the validity of the readings and were eager to understand whether the data could be trusted but had no direct means of doing so. For example, Pilot Program Member 2 noted, “I don’t know how they’re calibrating. I don't know how they’re calculating that.”

In retrospect, the approach of minimizing participant effort was misguided. As mariculturists, our participants had technical background and experience working with diverse technologies, making them capable of managing recalibration and validating readings. Pilot Program Member 3 made a valuable suggestion, noting “[Future See Salt program members need] a little bit of basic background on water monitoring. A lot of people do it, a lot of people will be at least privy to the salinity they’re growing in. But not everybody’s going to have the scientific background. It might be a learning curve for some, but honestly, it’s pretty cut and dry.” Thus, in a future iteration of See Salt, we will provide participants with the equipment and training needed to maintain the sensors and validate measurements. For example, we now advocate for providing to each participant a handheld conductivity and temperature sensor that is kept in calibration and not deployed in the field. This way, they could collect spot measurements to compare to the continuous sensor, such that they could identify potential inaccuracies and need for recalibration or additional technical support. While we estimated that recalibration needed to occur biweekly, the ability to spot check measurements could reveal that calibration should occur more frequently. Though we do not have the data needed to provide recommendations on calibration and cleaning frequency, we suspect maintenance should occur on a weekly, not biweekly, basis, or even more frequently during periods of high primary productivity, as noted by Pilot Program Participant 3, “[Cleaning is] going to be pretty low profile this winter as far as keeping it up versus spring and summertime and barnacles and everything else is growing.” To facilitate recalibration by growers, calibration solutions should be provided in addition to handheld sensors, along with a detailed standard operating procedure on how to recalibrate the sensors and document pertinent information. Ideally, the procedure would also be provided as a how-to video that participants could routinely access (e.g., via QR code affixed to the monitoring unit directly). As part of the training process, having semi-regular visits by program leaders would also help to ensure best practices are being implemented and provide greater opportunities for participant engagement. To summarize, we recommend that community-based monitoring programs invest in the equipment and training needed for participants to be self-sufficient in data collection protocols. This may require more of a time investment for participants, potentially reducing the number of community members involved, but is necessary to improve data quality and value of the program overall.

To provide training, partnerships could be made with existing outreach and education groups. For example, local extension activities via the Sea Grant system could host training workshops, perhaps in conjunction with local community colleges that offer technical training to aquatic industry workers. Providing demonstrations at local community events and “how-to” videos accessible on the internet are other options to communicate best practices.

## Conclusions

There are many barriers to growing the geographic footprint over which continuous coastal monitoring information is collected, but partnering with those who work on the water can create win–win opportunities to fill data gaps and support end-user needs and interests. Here, we summarize a pilot program we launched in 2024 to collect salinity and water level data in partnership with oyster growers, and highlight lessons learned and recommendations for others who may similarly want to partner with aquatic industry workers to support monitoring efforts. Oyster growers proved to be excellent partners in building a community-based environmental monitoring program—their interest in the data for both the quality of their product as well as the ecosystem they rely on, access to monitoring sites, and strong practical knowledge of coastal systems greatly informed our ability to assess the viability of future efforts. While we partnered with oyster farmers, other maritime professionals who could be engaged in such efforts include fishermen, lifeguards, dock workers, military personnel (e.g., Navy and Coast Guard), park managers, and educators. Ultimately, by partnering with community members, we can increase data access while also empowering people to take part in advancing scientific understanding of the coastal waters on which they depend.

## Supplementary information

Below is the link to the electronic supplementary material.

ESM1 (DOCX 3.93 MB)

## Data Availability

No datasets were generated or analysed during the current study.

## References

[CR1] Alliance for Coastal Technologies. (2007). *Use of, satisfaction with, and requirements for in situ salinity sensors.* [14pp.]. Alliance for Coastal Technologies (ACT). 10.25607/OBP-335

[CR2] CoCoRaHS. (n.d.). *cocorahs - Community collaborative rain, hail & snow network*. Retrieved February 21, 2025, from https://www.cocorahs.org/

[CR3] Conrad, C. C., & Hilchey, K. G. (2011). A review of citizen science and community-based environmental monitoring: Issues and opportunities. *Environmental Monitoring and Assessment,**176*, 273–291. 10.1007/s10661-010-1582-5 20640506 10.1007/s10661-010-1582-5

[CR4] Conrads, P. A., & Darby, L. S. (2017). Development of a coastal drought index using salinity data. *Bulletin of the American Meteorological Society,**98*, 753–766. 10.1175/BAMS-D-15-00171.1

[CR5] Delauney, L., Compère, C., & Lehaitre, M. (2010). Biofouling protection for marine environmental sensors. *Ocean Science,**6*(2), 503–511. 10.5194/os-6-503-2010

[CR6] Evans, K. S., Athearn, K., Chen, X., Bell, K. P., & Johnson, T. (2016). Measuring the impact of pollution closures on commercial shellfish harvest: The case of soft-shell clams in Machias Bay, Maine. *Ocean & Coastal Management,**130*, 196–204. 10.1016/j.ocecoaman.2016.06.005

[CR7] Helton, A. M., Dennedy-Frank, P. J., Emanuel, R. E., et al. (2025). Over, under, and through: Hydrologic connectivity and the future of coastal landscape salinization. *Water Resources Research,**61*(7), e2024WR038720. 10.1029/2024WR038720

[CR8] Hummel, M. A., Berry, M. S., & Stacey, M. T. (2018). Sea level rise impacts on wastewater treatment systems along the U.S. coasts. *Earth’s Future,**6*(4), 622–633. 10.1002/2017EF000805

[CR9] Kennish, M. J. (2002). Environmental threats and environmental future of estuaries. *Environmental Conservation,**29*(1), 78–107. 10.1017/S0376892902000061

[CR10] Koren, K., & McGraw, C. M. (2023). Let’s talk about slime; Or why biofouling needs more attention in sensor science. *ACS Sensors,**8*(7), 2432–2439. 10.1021/acssensors.3c00961 37409449 10.1021/acssensors.3c00961

[CR11] La Peyre, M. K., Eberline, B. S., Soniat, T. M., & La Peyre, J. F. (2013). Differences in extreme low salinity timing and duration differentially affect eastern oyster (*Crassostrea virginica*) size class growth and mortality in Breton Sound, LA. *Estuarine, Coastal and Shelf Science,**135*, 146–157. 10.1016/j.ecss.2013.10.001

[CR12] Mercaldo-Allen, R., Clark, P., Liu, Y., Phillips, G., Redman, D., Auster, P. J., Estela, E., Milke, L., Verkade, A., & Rose, J. M. (2021). Exploring video and edna metabarcoding methods to assess oyster aquaculture cages as fish habitat. *Aquaculture Environment Interactions,**13*, 277–294. 10.3354/aei00408

[CR13] Mills, K., Kennish, M. J., & Moore, K. A. (2008). Research and monitoring components of the National Estuarine Research Reserve System. *Journal of Coastal Research,**10055*, 1–8. 10.2112/SI55-012.1

[CR14] Piecuch, C. G., & Hamlington, B. D. (2023). Yesterday’s high tide is today’s new normal. *Earth’s Future,**11*(8), Article e2023EF003774. 10.1029/2023EF003774

[CR15] Porter, D. E., Small, T., White, D., Fletcher, M., Norman, A., Swain, D., & Friedmann, J. (2004). Data management in support of environmental monitoring, research, and coastal management. *Journal of Coastal Research,**10045*, 9–16. 10.2112/SI45-009.1

[CR16] Reges, H. W., Doesken, N., Turner, J., Newman, N., Bergantino, A., & Schwalbe, Z. (2016). CoCoRaHS: The evolution and accomplishments of a volunteer rain gauge network. *Bulletin of the American Meteorological Society,**97*(10), 1831–1846. 10.1175/BAMS-D-14-00213.1

[CR17] Routhier, M. R., Curran, B. R., Carlson, C. H., & Goddard, T. A. (2024). Remote sensing and assessment of compound groundwater flooding using an end-to-end wireless environmental sensor network and data model at a coastal cultural heritage site in Portsmouth, NH. *Sensors (Basel),**24*(20), Article 6591. 10.3390/s24206591 39460072 10.3390/s24206591 PMC11511167

[CR18] Short, F. T., Coles, R. G., & Short, C. A. (2015). *SeagrassNet manual for scientific monitoring of seagrass habitat, worldwide edition*. University of New Hampshire Publication. https://www.seagrassnet.org/seagrass_manual_2015.pdf. Accessed 21 Feb 2025.

[CR19] Tansel, B., & Zhang, K. (2022). Effects of saltwater intrusion and sea level rise on aging and corrosion rates of iron pipes in water distribution and wastewater collection systems in coastal areas. *Journal of Environmental Management,**315*, Article 115153. 10.1016/j.jenvman.2022.115153 35500487 10.1016/j.jenvman.2022.115153

[CR20] Tully, K., Gedan, K., Epanchin-Niell, R., Strong, A., Bernhardt, E. S., BenDor, T., Mitchell, M., Kominoski, J., Jordan, T. E., Neubauer, S. C., & Weston, N. B. (2019). The invisible flood: The chemistry, ecology, and social implications of coastal saltwater intrusion. *BioScience,**69*(5), 368–378. 10.1093/biosci/biz027

