# Supplementary material for: See Salt: Recommendations for engaging oyster growers in community-based coastal monitoring programs
Source: Environ Monit Assess. 2025 Sep 11;197(10):1109. doi: 10.1007/s10661-025-14560-y (PMC12425839; doi:10.1007/s10661-025-14560-y)
Supplement: Supplementary file 1 — (DOCX 3.93 MB) [file 10661_2025_14560_MOESM1_ESM.docx]

**Article Title**: See Salt: Recommendations for Engaging Oyster Growers in Community-Based Coastal Monitoring Programs

**Journal Name:** Environmental Monitoring and Assessment

**Authors Names**: Natalie G. Nelson^1,2,^*, Marcelo Ardón^2,3^, Tal Ben-Horin^4^, Eric Herbst^5^, Whitney Knollenberg^6^, María Menchú-Maldonado^1^, Christopher L. Osburn^7^

Corresponding author, affiliation, email: Natalie Nelson, North Carolina State University, nnelson4@ncsu.edu

**Online Resource 1: See Salt Pilot Program Interview Protocol (IRB 27282)**

*Thanks so much for your time, I’m really looking forward to learning more about your experience with the See Salt pilot program.*

1. ﻿To get us started, tell me about your experience in oyster mariculture.
   1. How long have you been engaged in oyster mariculture?
   2. Please describe your operation
      1. How many acres?
      2. General description of lease environment – which body of water, nearby inlets/deltas
      3. What type of gear do you use on your lease?
      4. Where do you sell your oysters?
      5. What other activities do you engage in, in addition to mariculture – tours/visits, events, research, education?
   3. What do you find most rewarding about growing oysters?
   4. What aspect of oyster mariculture presents the greatest challenge for you?

*Those details will be really helpful in understanding your experience with See Salt, thanks for sharing them. These next questions will focus on your experience with gathering salinity data for the See Salt pilot program.*

Value of Salinity Data

1. Please explain why salinity data is valuable for your oyster mariculture operation.
2. What motivated you to be involved in the collection of salinity data through the *See Salt* program?

Listen for – access to data, accuracy of data, cost of equipment, financial/business benefits.

Assess their experiences with See Salt pilot program

1. What are 1 – 2 things you recommend that we continue doing as we try to expand the *See Salt* program? In other words, what do you think worked well in the pilot program?
2. What are 1 – 2 things you recommend that we improve as we try to expand the *See Salt* program?
3. What unanticipated challenges arose during your participation in the *See Salt* pilot program?
4. Describe your experience with using both types of monitors - the TIM version and the Onset version. To expand the *See Salt* program we’ll need to select one monitor for all participants to use.
   1. Which monitor would you recommend using in the future, and why?
5. Based on your experience with the *See Salt* pilot program, what should other oyster growers know before they commit to participating in *See Salt* as we expand the program?
6. Would you continue participating in the *See Salt* program as we expand it?
   1. If yes – what benefits do you see from continued participation?
   2. If no – what is it that would keep you from continued participation?

*Thanks for sharing your experiences with the See Salt pilot program. Next, I’d like to get your thoughts on what we should be thinking about as we try to expand the See Salt program. This expansion would entail recruiting more oyster growers to collect data using a protocol like what you used – but improved based upon the information you provide today.*

Data Sharing

1. Describe the data sharing arrangement that you would feel most comfortable with for *See Salt*.
   1. Who should have access to the data? Examples could include…
      1. Researchers
      2. NC Division of Marine Fisheries
      3. USDA Farm Service Agency (for insurance claims)
      4. Other oyster growers (we are considering a co-op model, where those who participate in the co-op have access to their data and other participants)
      5. The public / everyone
   2. What level of public data sharing would be most appropriate?
      1. Data are shared and the exact coordinates of the data collection location are shown (e.g., as a pin on a map with lat/long coordinates)
      2. Data are shared and shown as having been collected somewhere in a large area (e.g., as a large multi-square mile circle without disclosing the specific location within the circle where data were collected)
      3. Data are shared for the waterbody they were collected in (e.g., the data could be shared as “collected in Core Sound” or “collected in the Newport River Estuary”)
      4. Data are shared at the county level

Perceptions of opportunities for expansion of the project

1. If we are able to expand *See Salt* into a user-generated data network that provides access to real time salinity data, how would the North Carolina oyster mariculture industry benefit?
2. Who else would benefit from having access to salinity data like that generated by *See Salt*?
   1. If oyster growers could provide the salinity data those groups benefit from, how might that impact the relationship those groups have with oyster growers?
3. What ways could you envision using salinity data, like that generated through *See Salt*, to educate the public?
   1. For example, how might you integrate data from *See Salt* into tours, events, educational materials, marketing materials, insurance claims?

Perceptions of challenges for expansion of the project

1. What challenges do you foresee in our efforts to expand *See Salt* to include more oyster growers in NC?
2. What resources would oyster growers need to participate in *See Salt?*

Listen for – equipment, training, funding, data access

*Thank you so much for sharing your thoughts on See Salt. You’ll be on our list to share updates about the program and our next steps. Do you have any questions for me?*
